# The Association between Body Mass Index and Vitamin D Supplement Use among Adults in the United States

**DOI:** 10.7759/cureus.5721

**Published:** 2019-09-21

**Authors:** Carlos Orces

**Affiliations:** 1 Rheumatology, Laredo Medical Center, Laredo, USA

**Keywords:** obesity, vitamin d, supplements

## Abstract

Background

Obesity has been consistently associated with lower 25-hydroxyvitamin D (25(OH)D) concentrations. BMI status also has a major impact on 25(OH)D levels among vitamin D supplement users. In our cross-sectional study, we aimed to discuss the prevalence of vitamin D supplement use and its effect on 25(OH)D levels according to BMI status among US adults.

Methods

The present analysis was based on data from the National Health and Nutrition Examination Survey (NHANES) cycles 2011-2012 through 2013-2014. The prevalence of vitamin D supplement use according to BMI status among subjects aged 20 years and older by selected characteristics was analyzed. Logistic regression models were assembled to examine the independent association of BMI status and vitamin D supplement use. Similarly, general linear models were used to assess the effect of daily vitamin D supplementation doses (<400 IU, 400-800 IU, and >800 IU) on 25(OH)D concentrations according to BMI status.

Results

Of 10,076 participants with a mean age of 47.2 [standard error (SE): 0.4] years, 57.5% (SE: 1.7) of normal-weight and 63.5% (SE: 0.9) of obese subjects reported not taking vitamin D supplements over the previous 30 days. After adjustment for potential confounders, obese subjects were 1.3 times more likely to be nonusers of vitamin D supplements and were 24% less likely to take vitamin D supplements ≥400 IU/day compared with their normal-weight counterparts. In addition, significant differences in mean 25(OH)D levels of vitamin D supplementation doses were consistently seen across BMI categories. Indeed, obese and overweight participants taking >800 IU/day of vitamin D supplements had 15.5 and 8.1 nmol/L lower mean concentrations of 25(OH)D than their normal-weight counterparts on average, respectively.

Conclusion

Obese subjects had a lower prevalence of vitamin D supplement use compared with their normal-weight counterparts. This nutritional disparity may also contribute to low 25(OH)D concentrations seen in obesity. However, a daily vitamin D supplement intake between 400 IU and 800 IU appears to be adequate among obese subjects to achieve optimal 25(OH) levels.

## Introduction

Lower serum 25-hydroxyvitamin D (25(OH)D) concentrations have been consistently reported across different latitudes in obese subjects compared with their normal-weight counterparts [1-9]. Similarly, interventional studies and randomized controlled trials (RCTs) of variable duration and vitamin D supplement doses have demonstrated an inverse 25(OH)D response according to BMI status and body fat [10-12]. Although the precise mechanism of low 25(OH)D levels in obese subjects has not been fully elucidated, previous studies have concluded that limited sun exposure, decreased bioavailability of vitamin D in fat tissue, or simply volumetric dilution may explain this association [4-6]. 

Notably, a recent analysis of the National Health and Nutrition Examination Survey (NHANES) demonstrated that obese older adults taking vitamin D supplements between 400 and 800 IU or >800 IU/day achieved 25(OH)D concentrations defined as sufficient [13]. However, Agarwal et al. described that more than 90% of US adults consumed less than the estimated average requirements of vitamin D. In addition, dietary intake of vitamin D was significantly lower among the obese compared with their normal-weight counterparts. Agarwal et al. also observed a lower prevalence of vitamin D inadequacy among vitamin D supplement users compared to nonusers, which applies to obese subjects as well [14]. Despite this evidence, there are scarce population-based data regarding the relationship between BMI status and vitamin D supplement use. Our cross-sectional study aimed to address this and examine the prevalence of vitamin D supplement use and its effect on 25(OH)D levels according to BMI status in a nationally representative sample of adults.

## Materials and methods

NHANES is a biannual cross-sectional study conducted by the National Center for Health Statistics (NCHS) of the Centers for Disease Control and Prevention (CDC). The purpose of NHANES is to collect data about the health, nutritional status, and health behaviors of the noninstitutionalized civilian resident population of the US. The NHANES data were obtained using a complex, multistage probability sampling design to select a sample representative of the US civilian household population [15]. In this analysis, 11,329 participants aged 20 years and older were selected for the NHANES cycles 2011-2014. Those participants who had missing data on BMI (n = 572), vitamin D supplements (n = 214), or 25(OH)D levels (n = 1,002) were excluded, leaving a total study sample of 10,076 participants. Overall, subjects with missing data were more likely to be older, non-Hispanic blacks, with a college education, non-smokers, and physically inactive. They were mostly reported to be in good-to-excellent health. 

Characteristics of participants

The demographics file provides individual, family, and household-level information on the following topics: the six-month time period when the examination was performed (November 1 through April 30 and May 1 through October 31), age, gender, race/ethnicity (Mexican-American and other Hispanic were grouped as Hispanic, non-Hispanic white, non-Hispanic black, and other race), education (<high school, high school/GED equivalent, some college or AA degree, and college graduate or above). The ratio of the family-income-to-poverty threshold as a measure of socioeconomic status was calculated, and families with a ratio of <1.00 were considered below the poverty level. Participants were also asked the following query: “Is there a place that you usually go to when you are sick or need advice about your health?”. Those who responded affirmatively to this question were considered to have access to health care. Moreover, a physical-activity questionnaire was used to assess participants’ leisure-time physical-activity status. The reported number of days and time in minutes spent performing vigorous or moderate leisure-time physical activity in the previous week were calculated. Based on the 2008 Physical Activity Guidelines for Americans, three levels of physical activity were created: 1) participants who engaged in ≥150 min/week of moderate activity, or ≥75 min/week of vigorous activity, or ≥150 mins/week of an equivalent combination were defined as physically active; 2) those who reported some physical activity but not enough to meet the active definition (>0 to <150 min/week) were considered insufficiently active; 3) those who reported no physical activity were considered inactive [16]. 

In the mobile examination center, BMI was calculated as body weight (kg) divided by height (m^2^). Subjects were classified as normal-weight if they had a BMI of <25.0 kg/m^2^, overweight if the BMI was 25-29.9 kg/m^2^, or obese in cases where BMI was ≥30 kg/m^2^. Since underweight subjects accounted for a small number of participants (n = 171), they were grouped with the normal-weight participants. Participants also reported their smoking status and were classified as "current", "former, or "never" smokers. Self-reported general health condition was grouped as "good to excellent" or "fair to poor". 

Vitamin D supplement use

The NHANES total dietary supplement file was used to examine the use of vitamin D supplements over the previous 30 days. All participants in the household sample were included in this file, including dietary-supplement users and non-users. Each total intake record contains the total number of supplements and antacids reported for that participant and the mean daily intake aggregates of 34 nutrients/dietary components from all supplements and antacids, as calculated using the NHANES dietary supplement database. Data were routinely examined for discrepancies and erroneous entries. Trained nutritionists reviewed incoming data and matched reported dietary-supplement entries to known supplements from the in-house product label database, where possible; sought additional product labels if feasible; assigned generic or default supplements as appropriate; transferred or removed products that were not considered dietary supplements; and assigned matching codes [17]. 

25-hydroxyvitamin D concentrations

The CDC-standardized liquid chromatography-tandem mass spectrometry (LC-MS/MS) method was used for measurement of 25(OH)D for NHANES 2011-2014, which allows laboratories and surveys to compare 25(OH)D measurements. CDC had decided to develop an LC-MS/MS method traceable to the NIST-reference materials for NHANES and had started using this method with NHANES 2007-2008. CDC recommends using the total 25(OH)D in SI units (nmol/L) measured directly by LC-MS/MS and converting this quantity to conventional units (1 nmol/L = 0.4066 ng/mL) if needed. This method has better analytical specificity and sensitivity compared to immunoassay methods and fixed analytical goals for imprecision (≤10%) and bias (≤5%) [18].

Statistical analysis

The descriptive characteristic of the study population stratified according to BMI status was reported as the percentage and mean values with their respective SEs. The chi-squared test was used to compare the proportion of participants taking vitamin D supplements stratified by BMI status across selected demographic, behavioral, and health characteristics. Subsequently, multiple logistic regression models were assembled to estimate the independent association of BMI status and vitamin D supplementation doses (none, ≥400 IU, and ≥800 IU) adjusted for six-month study period, age, gender, race/ethnicity, education, income, smoking habit, physical activity, health status, and access to health care. Similarly, in a subgroup analysis, adjusted general linear models were created to examine the effect of vitamin D supplement doses stratified according to BMI status on 25(OH)D concentrations. Statistical analyses were performed using SPSS Complex Sample software, V.17 (IBM, Armonk, New York) to incorporate constructed weights for the combined survey cycles and obtain unbiased, national estimates representative of the US population. A probability value (P) of <.05 was considered statistically significant. 

## Results

The study sample comprised a total of 10,076 participants with a mean age of 47.2 (SE: 0.4) years. The table below (Table 1) shows the characteristics of participants stratified by BMI status. Overall, a higher proportion of participants were middle-aged, females, non-Hispanic whites, had less than a high-school education, were physically inactive, and reported good-to-excellent health. 

**Table 1:** Characteristics of participants according to BMI categories, NHANES 2011-2014 SE: standard error; AA: associates of arts: RIP: ratio of family income to poverty

	Normal weight (n = 3,096), % (SE)	Overweight (n= 3,246), % (SE)	Obese (n= 3,734), % SE
Six-month period			
Nov 1-Apr 30	43.4 (4.3)	44.2 (4.7)	47.4 (4.6)
May 1-Oct 31	56.6 (4.3)	55.8 (4.7)	52.6 (4.6)
Age (years)			
20-39	45.7 (2.0)	32.7 (1.1)	32.2 (1.4)
40-59	31.3 (1.6)	39.2 (1.2)	41.6 (0.9)
≥60	23.0 (1.4)	28.1 (1.1)	26.2 (0.9)
Gender			
Male	43.0 (0.9)	55.4 (0.9)	45.9 (0.9)
Female	57.0 (0.9)	44.6 (0.9)	54.1 (0.9)
Race/ethnicity			
Hispanic	10.7 (1.3)	15.2 (1.8)	16.7 (2.1)
Non-Hispanic white	67.5 (2.3)	69.3 (2.5)	64.5 (3.0)
Non-Hispanic black	8.7 (1.2)	9.0 (1.1)	14.5 (1.9)
Other races	13.1 (1.1)	6.6 (0.6)	4.3 (0.5)
Education			
Less than high school	24.0 (2.1)	26.0 (1.4)	29.3 (1.4)
High-school graduate	22.7 (1.3)	25.7 (1.1)	30.6 (1.0)
Some college or AA degree	34.7 (2.3)	31.4 (1.5)	28.8 (1.0)
College graduate or above	18.6 (2.4)	16.9 (1.7)	11.4 (1.1)
RIP			
<1.00	17.7 (1.6)	14.1 (1.0)	17.0 (1.4)
≥1.00	82.3 (1.6)	85.9 (1.0)	83.0 (1.4)
Smoking status			
Never	57.1 (1.7)	56.2 (1.1)	55.7 (1.3)
Former	19.0 (1.1)	26.4 (1.1)	26.4 (1.2)
Current	24.0 (1.8)	17.4 (0.7)	17.9 (0.8)
Physical-activity status			
Inactive	37.7 (1.9)	43.5 (1.4)	54.7 (1.2)
<150 min/week	15.9 (1.0)	15.7 (0.8)	17.3 (0.8)
≥150 min/week	46.3 (1.9)	40.8 (1.4)	27.9 (1.1)
General health condition			
Good to excellent	87.4 (1.4)	85.9 (0.8)	75.8 (0.9)
Fair to poor	12.6 (1.4)	14.1 (0.8)	24.2 (0.9)
Place for health care			
Yes	80.9 (1.0)	84.8 (0.9)	87.4 (0.7)
No	19.1 (1.0)	15.2 (0.9)	12.6 (0.7)

**Figure 1:**
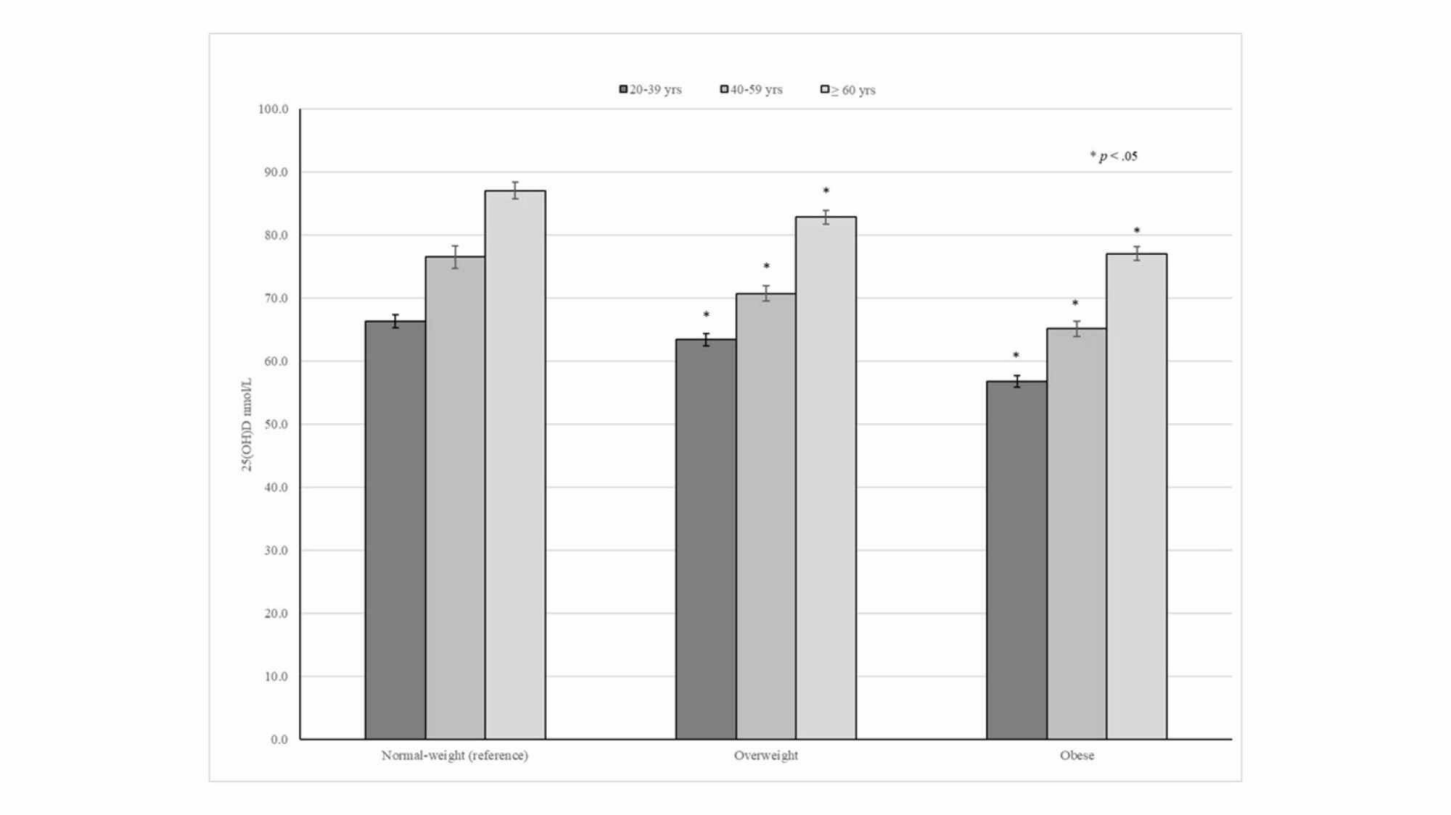
25(OH)D concentrations according to age groups and BMI status

In general, higher 25(OH)D concentrations were consistently seen among older adults independently of their BMI status. In addition, 25(OH)D concentrations across age groups progressively decreased in overweight and obese subjects (Figure 1). 

 

The table below (Table 2) shows the prevalence of vitamin D supplement use stratified by BMI status. Significantly, six-month time period during the fall and winter months, young and middle-aged subjects, women, non-Hispanic whites, household incomes above poverty level, physical activity, good-to-excellent health, and access to health care were characteristics associated significantly with a lower prevalence of vitamin D supplement use among obese subjects compared with their non-obese counterparts. 

**Table 2:** Prevalence of vitamin D supplement use according to BMI categories, NHANES 2011-2014 SE: standard error; AA: associates of arts; RIP: ratio of family income to poverty *P: <0.001; **P: <0.05

	Normal weight (n = 3,096), % (SE)	Overweight (n= 3,246), % (SE)	Obese (n= 3,734), % (SE)
Six-month period			
Nov 1-Apr 30	39.3 (2.2)	41.2 (1.8)	32.6 (1.6)*
May 1-Oct 31	45.1 (2.3)	45.2 (1.8)	40.0 (1.1)
Age (years)			
20-39	30.6 (2.3)	32.8 (2.0)	25.2 (1.3)**
40-59	46.6 (3.1)	42.7 (2.6)	35.4 (1.9)**
≥60	60.8 (2.6)	56.6 (2.6)	52.3 (2.6)
Gender			
Male	32.1 (1.6)	38.5 (1.5)	31.2 (1.4)*
Female	50.4 (2.1)	49.5 (1.8)	41.0 (1.4)*
Race/ethnicity			
Hispanic	24.4 (2.6)	23.5 (1.9)	24.2 (1.8)
Non-Hispanic white	48.5 (2.3)	49.4 (2.8)	41.3 (1.1)*
Non-Hispanic black	23.2 (2.7)	30.6 (2.7)	31.1 (1.0)**
Other races	39.7 (1.8)	43.6 (3.1)	31.3 (3.5)**
Education			
Less than high school	29.6 (1.7)	33.0 (2.5)	29.7 (1.9)
High-school graduate	33.0 (2.3)	40.4 (2.5)	36.7 (1.5)
Some college or AA degree	51.5 (3.1)	45.9 (1.8)	40.2 (2.1)**
College graduate or above	54.2 (2.8)	59.2 (3.6)	44.1 (2.4)**
RIP			
<1.00	22.5 (1.8)	24.3 (2.6)	22.8 (1.5)
≥1.00	47.4 (1.8)	47.4 (1.3)	39.5 (0.9)*
Smoking status			
Never	43.9 (2.1)	45.0 (1.9)	37.8 (1.4)**
Former	55.2 (2.9)	49.9 (2.5)	44.0 (2.2)**
Current	29.3 (2.8)	28.2 (2.3)	21.5 (1.6)**
Physical-activity status			
Inactive	35.0 (2.1)	36.7 (2.0)	34.2 (1.3)
<150 min/week	48.1 (2.6)	46.3 (1.8)	41.7 (2.4)
≥150 min/week	46.9 (2.2)	49.4 (2.1)	38.0 (1.8)*
General health condition			
Good to excellent	43.0 (1.8)	45.7 (1.4)	36.9 (0.9)*
Fair to poor	39.2 (2.8)	29.5 (2.1)	35.4 (2.2)**
Place for health care			
Yes	46.7 (1.9)	46.7 (1.4)	38.1 (1.0)*
No	25.0 (2.2)	24.7 (2.8)	25.3 (2.5)

Overall, 1,903 (57.5%), 1,989 (56.6%), and 2,443 (63.5%) of normal-weight, overweight, obese participants reported not taking vitamin D supplements over the previous 30 days, respectively. Moreover, only 28.1% of the obese subject reported taking vitamin D supplements ≥400 IU/day. After adjustment for potential confounders, obese participants were 1.3 times more likely to be nonusers of vitamin D supplements and were 24% less likely to use vitamin D supplements ≥ 400 IU/day compared with their normal-weight counterparts. Although obese participants were less likely to take vitamin D supplements ≥ 800 IU/day, differences across BMI categories were not statistically significant (Table 3). 

**Table 3:** Association of BMI status and vitamin D supplement use OR: odds ratio; CI: confidence interval ^a^Models adjusted for the six-month study period, age, gender, race-ethnicity, education, income, smoking status, physical activity, health status, and access to health care *p: <0.05

	Normal weight (n= 3,096)	Overweight (n= 3,246)	Obese (n= 3,734)
No vitamin D supplement			
% (SE)	57.5 (1.7)	56.6 (1.3)	63.5 (0.9)*
Crude OR (95% CI)	1.00 (ref)	0.96 (0.82, 1.13)	1.28 (1.10, 1.50)
Adjusted OR (95% CI)^a^	1.00 (ref)	0.99 (0.84, 1.7)	1.30 (1.10, 1.54)
Vitamin D ≥400 IU/day			
% (SE)	32.9 (1.5)	32.7 (1.3)	28.1 (0.9)*
Crude OR (95% CI)	1.00 (ref)	0.99 (0.84, 1.15)	0.79 (0.68, 0.91)
Adjusted OR (95% CI)^a^	1.00 (ref)	0.93 (0.79, 1.09)	0.76 (0.64, 0.91)
Vitamin D ≥800 IU/day			
% (SE)	18.8 (1.5)	18.5 (1.1)	16.4 (0.8)
OR (95% CI)	1.00 (ref)	0.98 (0.82, 1.16)	0.84 (0.69, 1.03)
Adjusted OR (95% CI)^a^	1.00 (ref)	1.01 (0.81, 1.26)	0.86 (0.65, 1.13)

**Figure 2:**
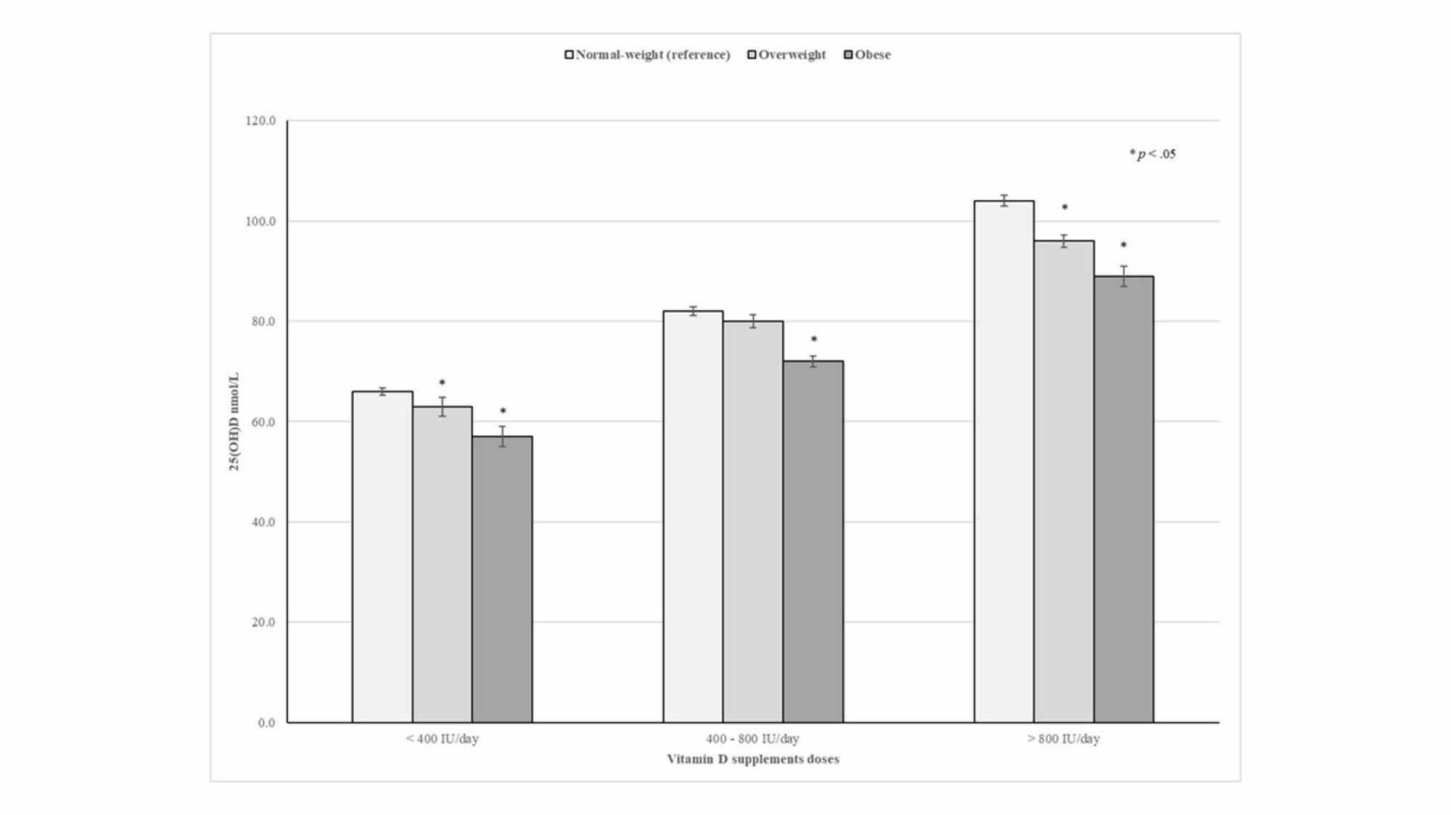
The effect of vitamin D supplements on 25(OH)D concentrations according to BMI status

Notably, mean 25(OH)D levels progressively increased as vitamin D supplementation doses increased. However, significant differences in mean 25(OH)D levels according to vitamin D supplementation doses were consistently seen across BMI categories. In fact, obese and overweight participants taking vitamin D supplements >800 IU/day had 15.5 and 8.1 nmol/L lower mean concentrations of 25(OH)D than their normal-weight counterparts on average, respectively (Figure 2). 

## Discussion

In this nationally representative sample of adults, vitamin D supplement use significantly differed according to BMI status. Overall, the use of vitamin D supplements was consistently lower among obese subjects compared with their normal-weight counterparts. This nutritional disparity was particularly seen among young and middle-aged participants, females, non-Hispanic blacks, subjects with a college education, and those with access to health care. Previous studies have also reported a higher risk of inadequate intake for several key micronutrients and dietary quality among obese adults compared to adults with a healthy weight [14,19-20]. Notably, the present findings indicate that only one-third of US adults met the 2011 Institute of Medicine (IOM) estimated average requirement for vitamin D intake (≥400 IU/day) from dietary supplements during the study period [21]. Moreover, after adjustment for potential confounders, obese subjects were 24% less likely to report taking vitamin D supplements ≥400 IU/day than their normal-weight counterparts. Recently, Blumberg et al. reported that among US adults, the use of multi-vitamin at any frequency significantly increased nutrient intakes and decreased the percentage of the population with inadequate intakes for most micronutrients, as compared with food alone [22]. Blumberg et al. also observed that the prevalence of vitamin D intake inadequacy decreased from 92.5% with food-only to 17.3% when vitamin D supplements were added. In addition, as compared with nonusers of vitamin D supplements, participants who reported taking vitamin D supplements 21 or more days decreased the odds of having vitamin D deficiency by 76% [22]. 

Several studies have shown that BMI is a major determinant of 25(OH)D concentrations following vitamin D supplementation [11-12,23-24]. In fact, a placebo-controlled trial of vitamin D supplementation conducted among postmenopausal white women with vitamin D insufficiency demonstrated that overweight and obese women had lower levels of 25(OH)D concentration (about 12.5 nmol/L and 17.5 nmol/L, respectively) compared with women with normal-weight at 12 months regardless of whether the vitamin D dose was administered or not [24]. Likewise, a large study among healthy volunteers in Alberta, Canada, reported that 600 IU/day of vitamin D supplementation achieved an average 25(OH)D concentration of 83, 76 and, 66 nmol/L in normal-weight, overweight, and obese participants, respectively [25]. The present results also demonstrate that 25(OH)D concentrations differed according to BMI status among vitamin D supplement users. Indeed, even after adjustment for potential confounders, normal-weight, overweight, and obese participants who reported taking vitamin D supplements between 400 and 800IU/day had mean 25(OH)D concentrations at 82, 80, and 72 nmol/L, respectively. 

Obese subjects who use vitamin D supplements between 400 and 800IU/day or higher doses had mean 25(OH)D concentrations consistently higher than 50 nmol/L, which is the 25(OH)D cutoff level defined as sufficient by the IOM report [21]. Although, the Endocrine Society has recommended that obese subjects may need at least two or three more vitamin D intakes (at least 6,000 to 10,000 IU/day) to treat or prevent vitamin D deficiency, the present findings demonstrate that obese subjects who reported taking vitamin D supplements between 400 and 800 IU/day were able to reach optimal 25(OH)D concentrations (≥50 nmol/L). Therefore, the IOM-recommended vitamin D dietary allowance of 600 IU/day for subjects aged 20-70 years and 800 IU/day for those >70 years should be considered adequate for obese adults until further research evidence is available. 

Significantly, studies conducted to analyze the dose-response of vitamin D supplements on 25(OH)D concentrations have consistently reported parallel curves across BMI status, suggesting that the differences in 25(OH)D concentrations are most likely due to a volume dilution effect, which is consistent with the present findings [5,11,24]. Moreover, Gallagher et al. reported a small difference in dose-response curves of vitamin D supplements on 25(OH)D levels between normal-weight and obese subjects, suggesting that inadequate intake of vitamin D may also account for lower 25(OH)D levels seen in obesity [11]. 

Interestingly, young adults had a lower prevalence of vitamin D supplement use compared with their middle-aged and older adult counterparts, which was also more marked in obese participants. Similarly, young adults had lower 25(OH)D concentrations across BMI status compared with those in other age groups. In fact, the lowest 25(OH)D concentration at 56.8 nmol/L was seen among obese adults aged 20-39 years. Thus, these findings suggest that obese young adults might be at increased risk of vitamin D inadequacy and should be counseled about vitamin D supplementation. Although the effect of low 25(OH)D concentrations among obese subjects on bone mineralization is not very clear, a recent nationwide cross-sectional analysis demonstrated that BMI was positively associated with femoral-neck-bone mineral density (BMD) among adults aged 50 years and older, irrespective of their sex or race [26]. In contrast, a prospective study conducted among participants in the Health ABC study reported that obese older adults had significantly lower mean femoral-neck BMD than those with normal weight at 10 years of follow-up [27].

Several limitations should be considered while interpreting the study results. First, because of the NHANES cross-sectional design, the present findings do not necessarily infer causation. Second, sun exposure and sun-protective behavior variables, which may affect the synthesis of 25(OH)D3, were not explored in the present analysis. Third, the effect of latitude on participants’ 25(OH)D concentrations was unknown. Fourth, most sociodemographic variables were self-reported, which may have led to recall bias. Finally, although there are few naturally occurring food sources of vitamin D, dietary vitamin D was not examined in this study. However, dietary vitamin D may also contribute to 25(OH)D concentrations [21].

## Conclusions

Our study concluded that obese subjects had a lower prevalence of vitamin D supplement use compared with their normal-weight counterparts. This nutritional disparity may also contribute to low 25(OH)D concentrations seen in obesity. However, a daily vitamin D supplement intake between 400 IU and 800 IU appears to be adequate among obese subjects to achieve optimal 25(OH) levels. 
